# How Machine Learning Methods Helped Find Putative Rye Wax Genes Among GBS Data

**DOI:** 10.3390/ijms21207501

**Published:** 2020-10-12

**Authors:** Magdalena Góralska, Jan Bińkowski, Natalia Lenarczyk, Anna Bienias, Agnieszka Grądzielewska, Ilona Czyczyło-Mysza, Kamila Kapłoniak, Stefan Stojałowski, Beata Myśków

**Affiliations:** 1 Department of Plant Genetics, Breeding and Biotechnology, West-Pomeranian University of Technology, Szczecin, ul. Słowackiego 17, 71–434 Szczecin, Poland; Magdalena.Goralska@zut.edu.pl (M.G.); jan.binkowski96@gmail.com (J.B.); ln21018@zut.edu.pl (N.L.); anna.bienias@zut.edu.pl (A.B.); stefan.stojalowski@zut.edu.pl (S.S.); 2 Institute of Plant Genetics, Breeding and Biotechnology, University of Life Sciences in Lublin, ul. Akademicka, 20–950 Lublin, Poland; agnieszka.gradzielewska@up.lublin.pl; 3 Polish Academy of Sciences, The Franciszek Górski Institute of Plant Physiology, Niezapominajek 21, 30–239 Kraków, Poland; i.czyczylo@ifr-pan.edu.pl (I.C.-M.); kaploniak@ifr-pan.edu.pl (K.K.)

**Keywords:** ATP-binding cassette (ABC) transporters, fatty acid desaturase (FAD), genetic map, glaucousness, large-scale sequence-based markers, *Secale cereale* L.

## Abstract

The standard approach to genetic mapping was supplemented by machine learning (ML) to establish the location of the rye gene associated with epicuticular wax formation (glaucous phenotype). Over 180 plants of the biparental F_2_ population were genotyped with the DArTseq (sequencing-based diversity array technology). A maximum likelihood (MLH) algorithm (JoinMap 5.0) and three ML algorithms: logistic regression (LR), random forest and extreme gradient boosted trees (XGBoost), were used to select markers closely linked to the gene encoding wax layer. The allele conditioning the nonglaucous appearance of plants, derived from the cultivar Karlikovaja Zelenostebelnaja, was mapped at the chromosome 2R, which is the first report on this localization. The DNA sequence of DArT-Silico 3585843, closely linked to wax segregation detected by using ML methods, was indicated as one of the candidates controlling the studied trait. The putative gene encodes the ABCG11 transporter.

## 1. Introduction

**Figure 1:**
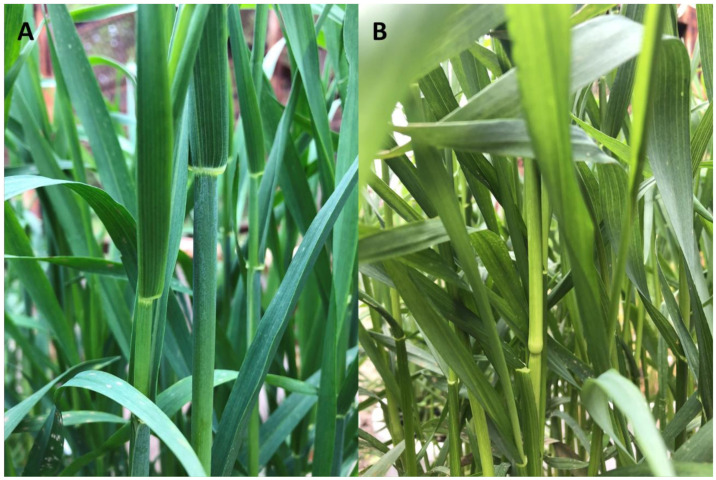
Stems and leaves of parental components of rye mapping population BK2: glaucous inbred line AK1 (**A**) and nonglaucous AKZ (**B**).

Objective of the StudyThe main aim of the study was to establish the localization of an unknown rye gene causing the waxless plant character (Figure 1) on a novel, high density genetic map constructed with DArTseq (sequencing-based diversity array technology) used for genotyping the biparental F_2_ population. The research was based on the standard approach to linkage genetic mapping methods in combination with ML (three algorithms) to find markers closely linked to the gene of interest (putative candidate genes).

Plant WaxesThe surface of primary above-ground plant organs is covered with a cuticle, a protective lipid structure sealing the tissues and insulating them from the effects of various unfavorable environmental factors. The cuticle serves as the major barrier preventing nonstomatal water loss and helps to protect plant surfaces from pathogens and ultraviolet radiation [1]. Plant cuticles consist of cutin, an insoluble polyester polymer lattice, and of soluble cuticular waxes. The waxes are either deposited within the cutin matrix (intracuticular wax) or accumulate on its surface as epicuticular wax [2].

The external appearance of plants is largely dependent on epicuticular wax structures, which are usually deposited on the plant’s surfaces as crystalloid forms or films. The bluish color of plant organs, caused by the scattering of light by wax crystals, is referred to as glaucousness, and the opposite form is referred to as nonglaucous, waxless or glossy [3]. The thickness of epicuticular wax layers, their composition and the shape of wax crystals/microstructures depend on the species [4], developmental stage and anatomical location [5,6]. In addition, the presence of wax is modified by environmental conditions [7].

The chemical composition of wax is very complex. In most cases, the majority of compounds comprising the cuticular wax are derived from very long-chain fatty acids (VLCFAs; C20–C34), including alkanes, aldehydes, primary and secondary alcohols, ketones and esters [2]. Waxy epicuticular layers in cereals also contain others various hydrophobic substances including alkyl and alkenyl resorcinols [8].

Moreover, since the lipid precursors of the cuticle are synthesized within epidermal cells and must pass through the hydrophilic cell wall to the developing cuticle, molecules carrying these compounds play an important role in cuticle/wax secretion. The group of proteins responsible for the transport of both wax and cutin precursors across the plasma membrane has been shown to depend on ATP-binding cassette (ABC) transporters [2].

Wax GenesThe presence of epicuticular wax crystals, which give the plant a glaucous appearance, has provided an easy way to detect mutants with wax crystal formation disorders. Such mutants, called eceriferum (cer), bloomless (bm) and glossy (gl), were the material for numerous molecular analyses, which led to an increasingly complete view of the wax biosynthesis pathway [2].

Using genetic methods, many wax genes, such as *CER1* [9], *CER2* [10], *CER3* [11], *CER4* [12], *CER5* [13], *CER6* [14,15], *CER7* [16], *CER8* [17], *CER9* [18,19] and *CER10* [20,21] have been identified in Arabidopsis and well characterized at the molecular level. Nonglaucous mutants of other species have also been tested quite intensively. The *BrWax1* and *BrCER4* genes in *Brassica rapa* and *BoWax1* in *B. oleracea*, that control the glossy phenotype, have been mapped [22,23,24]; the maize (*Zea mays*) GLOSSY1 (GL1) gene was molecularly cloned and characterized [25] and several rice genes encoding for the wax layer have also been studied [26]. Among the studies associated with wax synthesis in wheats, those regarding genes from chromosome group 2, controlling β-diketone production, predominate [3,27,28,29,30,31]. The barley *Cer-cqu* loci that determines β-diketone biosynthesis and glaucousness have also been studied [32,33].

Among the genes conditioning wax formation, those associated with the transport of wax precursors belonging to the ABC family play an important role. Recent studies showed that members of the G subfamily are critical for FAs (fatty acids) export. *ABCG11*/*WBC11*/*DSO*, *ABCG12*/*CER5*, *ABCG13*, *ABCG32*/*PEC1* and *OsABCG31*/*HvABCG31*/*gl13* [13,34,35,36,37,38,39,40,41] were reported to be required for lipid export.

Rye WaxesRye (*Secale cereale* L.), belonging to the *Poaceae* family, is one of the important cereal crops, grown mainly in the north-east part of Europe. Its grain is used for animal feed, alcohol production and bread flour. Rye has a number of positive attributes, such as outstanding cold hardiness, excellent drought tolerance and strong disease resistance. Some of these attributes may be due to the intense waxy bloom on the stem and leaf sheath. Few studies of the rye cuticle have concentrated on the chemical composition of cuticular waxes, [1,42]. The study on the total wax mixture from both sides of the leaves indicated the presence of primary alcohols (71%), alkyl esters (11%), aldehydes (5%) and small amounts (<3%) of alkanes, steroids, secondary alcohols, FAs and unknowns [1].

The genetic basis for creating the waxy epidermal coating is much less known. Only two genes responsible for the waxy film on straw, leaves and spikes have been localized on the genetic map of rye [43,44]. The recessive mutations determining the nonglaucous plant character have been mapped on chromosome 4R (*w*) and on the distal part of chromosome 7RL (*wa1*), which is translocated with respect to wheat and barley and is homologous to a distal region of the group 2 chromosomes (Rognli et al., 1992 in [43]). Both localizations are not very precise due to the low density of the applied genetic map.

Genetic MappingGenetic linkage mapping in segregating populations is one of the classic approaches to gain insights into the genetic control of key characteristics in a species. Continuous progress in the area of DNA technology, and powerful genotyping systems, have allowed the development of genomics, including genetic linkage mapping (www.maizegdb.org), [45,46,47,48,49,50,51]. Genetic maps are a source of valuable information for both cognitive genetics and practical applications. Markers linked to desirable genes or QTLs are important tools for improving plants in marker assisted breeding (MAB), via marker-assisted selection (MAS) and marker-assisted backcrossing (MABC). Map design is the first step towards positional cloning of genes responsible for a particular phenotype, with ultra-dense genetic maps required for this type of research [52,53].

### Rye Genetic Maps—Genes and QTL

The first genetic map of all seven rye chromosomes was created for over a quarter of a century ago [54] with RFLP (restriction fragment length polymorphism) and isozyme markers. Subsequent maps were constructed using the emerging marker techniques: PCR (polymerase chain reaction), microarrays and sequencing technologies, but their number is still smaller than in other important cereals. The first map created using high-throughput technology was constructed in 2011 [46]. The development of the transcript map was the next, important step in expanding the knowledge of the rye genome [55]. A consensus map of four populations included 2886 genes out of 3588 markers (80%). The map presented here is one of the few created with the use of GBS (genotyping by sequencing) markers [49,56,57]. DArTseq, like DArT, is a method in which genome complexity reduction is obtained using a combination of two restriction enzymes, at least one of which is methylation-sensitive, directing the analysis to the hypomethylated, gene-rich genome regions [49]. Compared to DArT, the new platform gives an increase in the number of available markers and provides codominant markers SNPs (single nucleotide polymorphisms), which improve the informativeness of the map. The abundance of markers, and the high probability of their localization in gene regions, make it possible to find markers closely related to an unknown gene as a good starting point for the search for potential candidate genes.

Previous rye maps have contributed significantly to the advancement of comparative and evolutionary research [54,55,58]. They were also used to find out the localization of genes responsible for, amongst other traits plant height/dwarfism [54,55,58,59,60,61], fertility restoration [62,63,64,65,66], rust resistance [67] and heading date [68], and were the basis to detect QTLs (quantitative trait loci) for different morphology/agronomic traits [69,70,71,72], alpha-amylase activity and preharvest sprouting [73,74,75].

Phenotypic glaucousness markers have so far been located only on older generation maps, poorly saturated with markers [43,44]. The recessive mutations determining the nonglaucous plant character was mapped on 4R (*w*) and 7RL (*wa1*).

The procedure used in this work consisted of constructing a highly saturated map using a moderate size mapping population, and selecting markers to create, in the future, a precise, fine map of the region with the gene responsible for a studied feature, using a larger mapping population. With such an approach it is important to select the right markers for further analysis, which can be problematic due to insufficient map precision. Genetic map design depends on many factors such as type and size of population, marker system and statistical method. It has been found that more accurate maps are obtained with F_2_-codominant and recombinant inbred lines than with backcrosses, double haploids and F_2_-dominant populations, and that a sample size of 200 individuals is sufficient for the construction of reasonably accurate maps [52].

Our experience with a population of less than 200 genotypes, and thousands of both dominant and codominant markers, shows that with such a large amount of data it is difficult to select the right set of loci to create proper linkage groups and to get the correct order of markers. To overcome this difficulty and improve marker selection, we decided to apply and test ML as a support method.

Machine Learning (ML) ApplicationsCollecting large amounts of data, as well as hardware and software progress in recent years, have contributed significantly to the increased use of ML in various fields of science. ML is becoming a powerful approach to data analysis, especially in human diagnostics and medicine. The early application of ML to population genetics demonstrated that it outperformed traditional approaches [76]. ML is becoming more and more common in plant genetics and breeding, mainly for genotype-phenotype relationship predictions and genomic selection. Researchers proposed ML algorithms as valuable alternatives to traditional statistical techniques applied in the breeding of maize [77], wheat [78] and soybean [79]. Due to advances in genomics, leading to the identification of numerous DNA markers, ML can be a complement to classical methods of genomic analysis.

## 2. Results

### 2.1. Effect of Lack of Wax Cover on Phenotypic Features in the Mapping Population

Decreased plant height and spikelet number per spike in two generations of the rye mapping population BK2 were observed in waxy plants (Table 1). Although there was no significant difference in length of the ear, the smaller number of spikelets resulted in a smaller density of ear (lower compactness) in this group of plants. Similar relationships were observed for two parental lines (data not shown). The difference in flowering date between glaucous and nonglaucous plants was also statistically significant, although small (Table 1).

### 2.2. Genotyping by Sequencing the BK2 Mapping Population with DArTseq Technology

The used GBS platform supplied 39,615 DArTseqs (28,889 Silico and 10,726 SNP). Among them 7,258 were rejected from further analysis because of missing data about the parental alleles or because of monomorphic character (Table 2). Most of the markers differentiating parental lines had an unknown genomic location.

### 2.3. Position of the Wax Locus in the Rye Genome Determined Using Mapping Procedure

Using the information from the GBS platform, based on localization of DNA sequences reported by Bauer et al. [57] and the publication on the dense genetic map of rye containing GBS markers [56], 8,135 (6,660 Silico and 1,475 SNP) were assigned to the seven respective chromosomes. Preliminary linkage analysis (grouping command) with these seven groups suggested localization of the *wax* locus on chromosome 2R (Table S1). From the set of loci unassigned to chromosomes, listed in Table S2 (24,222: 19,152 Silico and 5,070 SNP), those with which wax was linked were selected. As a selection criterion, the linkage at LOD (logarithm of odds) 50 was applied in the JoinMap 5.0 software (Kyazma B.V., Wageningen, The Netherlands). Further linkage analyses was performed on the group of DArTseqs assigned to chromosome 2R, showed in Table S1 (1135: 927 Silico and 208 SNP) combined with 698 DArTseqs selected from a group of loci with a previously unknown location.

The set of 1833 DArTseqs, and one wax segregation recorded in two ways (dominant and codominant), were subjected to grouping analysis (Table S3). Both the dominant type of wax layer segregation noted in F_2_ generation, and the codominant one (based on F_3_ generation), were used to control the mapping results correctness.

Grouping parameters were established at the LOD range from 10 to 50. At the lowest assumed level, 1756 of 1835 loci remained linked. LOD 36 was the maximum value at which both records of wax segregation remain associated in one group. The trial maps were created at LOD 36 (1670 loci), LOD 45 (982 loci) and LOD 50 (882 loci). As wax dominant segregation fell out of the group at highly restrictive values exceeding LOD 36, it was manually attached to the linkage groups created at LOD 45 and 50.

**Figure 2:**
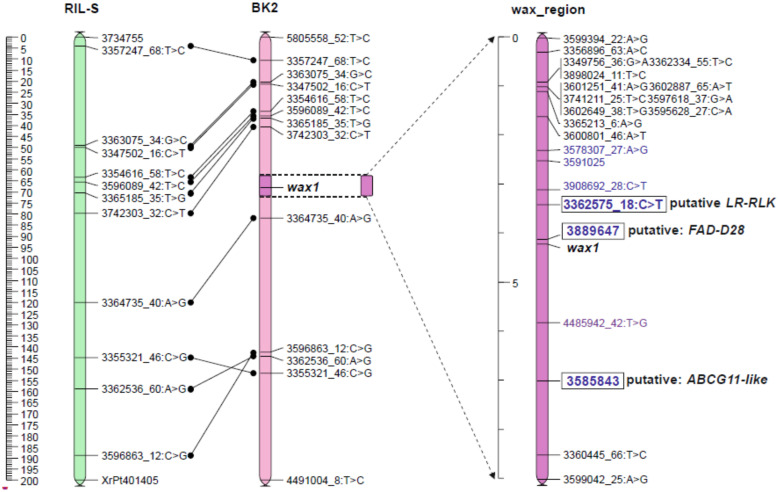
Position of the *wax* locus on the rye chromosome 2R of the BK2 mapping population with reference to the RIL-S map [56]. DArTseqs indicated in the ML analysis are blue.

The JoinMap 5.0 software gives two options for mapping procedure. The regression mapping algorithm (RM) simultaneously analyzes the relationships between several closely related loci, so this method is more accurate but more complex. When the number of loci is high, it requires very efficient computer equipment and is extremely time consuming. The alternative method, based on the maximum likelihood (MLH) procedure, considers relationships only between two neighbor markers in one step. This method does not allow removal of single errors in genotyping and usually gives over-estimated distances between loci but needs less effective computers and can be performed within hours, not weeks. Due to the large number of markers in linkage groups, the maps were constructed using the MLH algorithm. The maps created at LOD 36 and LOD 45 were not comparable to the reference map (Table S4). Common loci positions were strongly displaced, which made it impossible to determine the position of the wax locus. The map at LOD 50 had eleven common loci with a reference map of the RIL-S population [56], and their arrangement was mostly consistent (Figure 2). At this level, both wax segregations remained unseparated by other markers, as was the case with maps formed from loci grouped at lower LODs. This map (Table S5) was used to select loci nearest to the *wax* locus to map the *wax* region more precisely with these markers, and those detected by ML methods. The *wax* locus was located in the middle part of chromosome 2R (Figure 2).

### 2.4. Markers Distinguishing Between Waxy and Waxless Plants Detected Using Machine Learning (ML) Methods

To select markers that differentiate between waxy and waxless plants, three classifiers (LR, random forests and XGBClassifier) were trained. Each of the used algorithms assesses the features’ significance based on their impact on the classification process.

For training purpose, the dominant wax dominant segregation was used, dividing the plants into two classes. Two groups of DArTseqs were used independently, 1,135 assigned to chromosome 2R (group A) based on literature data [56,57], and 1067 linked to wax at LOD 45 selected from the group of markers previously not qualified for any chromosome (group B). Models prepared on the training dataset had strong predictive skills measured with classification metrics. Trained models had an F1 metric at the levels 97–98% for the glaucous class and 88–94% for the nonglaucous class. 

**Figure 3:**
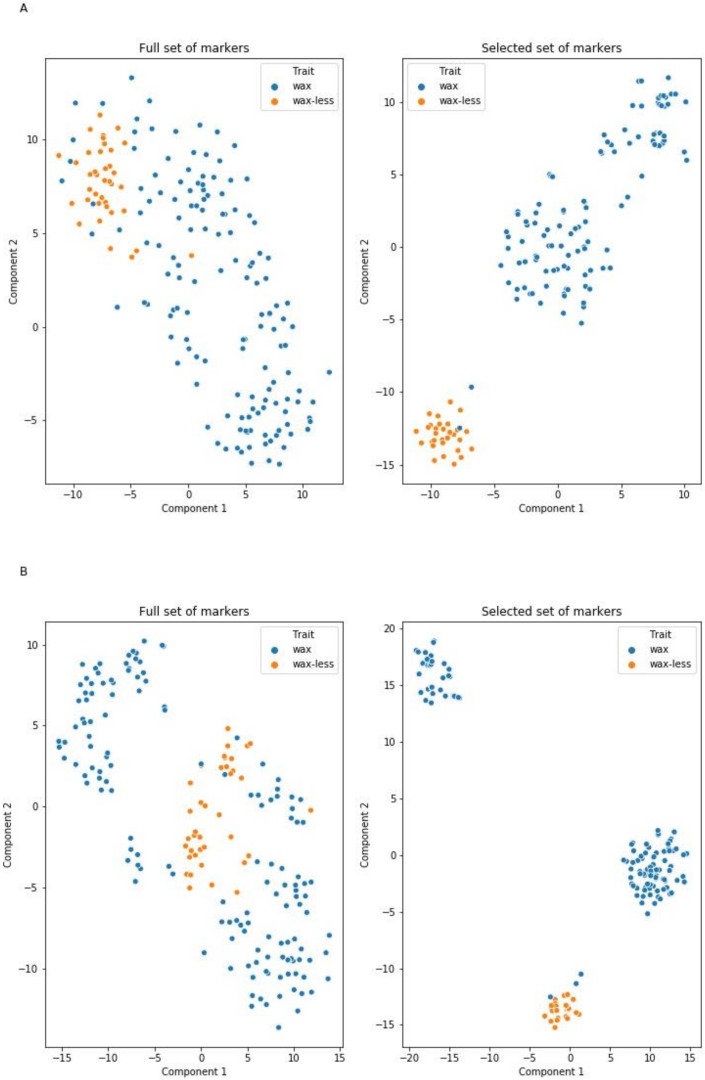
Projection of two classes of plants (wax and waxless) in a two-dimensional space characterized on the basis of DArTseqs assigned to chromosome 2R (**A**) and not assigned (**B**). The figures on the right show the ordering result using markers selected based on ML algorithms.

Marker selection to subset considered by models as informative allowed reduction of the number of markers from 1135 to 20 and from 1067 to 35 (Table S6). New models trained on the selected features matrix with the same hyperparameters had significantly higher metric values (F1-score at levels 98–99% for the glaucous class and 91–96% for the nonglaucous class). Furthermore, two-dimensional visualization of raw and selected DArTseqs performed using the T-distributed stochastic neighbor embedding (t-SNE) method shows that markers indicated by the models as informative form linearly separable groups representing opposing states of the feature (Figure 3). This observation, as well as increasing the models’ metrics, indicate that the ML-based selection process reduced noise resulting from the presence of irrelevant markers, and retained the most informative features from the original dataset allowing prediction of the type of phenotype (glaucous or nonglaucous) using 20 or 35 markers (models trained on dataset A and B, respectively) with 97–99% general prediction accuracy.

The most important markers appointed in the classical mapping procedure (MLH, LOD 50), and chosen by three ML models, were used to map the *wax* region. A group of 17 loci flanking *wax* on the map of the whole chromosome 2R (Table S5), 13 markers detected by all three ML methods (Table 3), and two records of wax segregation, were directed to the mapping procedure. A total of 31 loci were used because one DArTseq-SNP, 3362575_18:C>T, was common to both modes of selection. Using JoinMap 5.0 and the RM algorithm, a 30 cM map (Table S7) was created from which approximately a 10 cM interval was shown (Figure 2).

### 2.5. Genetic Map of Wax Region

#### 2.5.1. Candidate Genes for Wax Layer Creation

Sequences of DArTseqs (Table S3) linked to *wax* were directed to an NCBI-BLAST (The National Centrum for Biotechnology Information—Basic Local Alignment Tool) algorithm to find homologues. The marker most strongly linked to *wax*, DArT-Silico 3889647, was homologous to mRNA of the gene involved in long chain FA metabolism (Table 4). The second locus (3362575_18:C>T) nearest to *wax* was probably a gene for inactive leucine-rich repeat receptor-like protein kinase (LRR-RLK). The next DArT-Silico 3585843 strongly linked to *wax* was homologous to mRNA for the ABC transporter G family (*ABCG11-like*). Both DArT-Silico markers were selected using three ML methods, and DArT-SNP was appointed by both ML and classical mapping procedure (MLH). The sequence of DArTseq 4485942_42:T>G located between *wax* and *ABCG11-like* had no annotation found.

#### 2.5.2. Expression Analysis of Candidate Gene for ABCG11 Transporter

**Figure 4:**
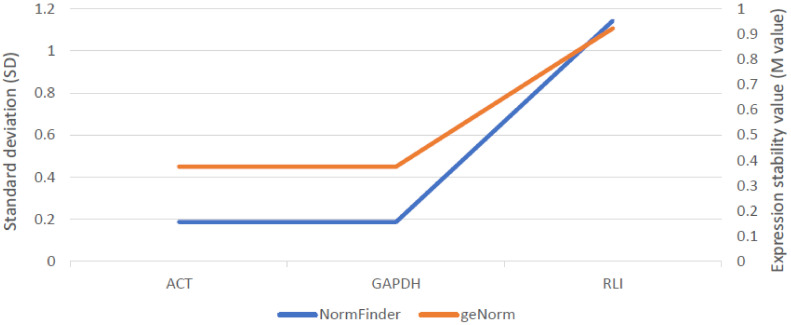
Stability assessment of three reference genes across all rye samples using two different algorithms: NormFinder (SD) and geNorm (*M*-Value) provided in GeneEx 7.0 software (bioMCC, Freising-Weihenstephan, Germany).

Among three examined reference genes, actin (ACT), glyceraldehyde 3-phosphate dehydrogenase (GAPDH) and RNase L inhibitor (RLI), expression of ACT and GADPH were the lowest and most stable across all samples. Therefore, both these genes were used as internal controls subsequently (Figure 4).

**Figure 5:**
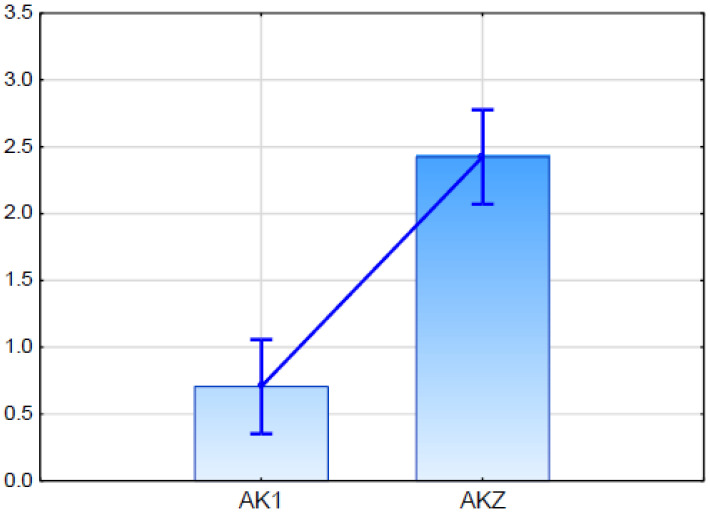
Relative expression (fold change in relation to the ACT and GAPDH) of putative *ScABCG11* established in qPCR (quantitative PCR) for glaucous (AK1) and nonglaucous (AKZ) rye inbred line. Vertical bars indicate +/− standard error. Differences are statistically significant in Kruskal-Wallis test.

Real Time PCR analysis demonstrated that the putative *ScABCG* was expressed in both studied rye lines. The expression level in glaucousness line AKZ was higher than in the glaucous line AK1, and the difference was statistically significant (Figure 5).

## 3. Discussion

Molecular analysis of wax-deficient mutants has led to the identification of a large number of genes encoding functional proteins in the biosynthesis, transport and regulation of cuticular wax in different plant species. Many elements of the cuticle and wax biosynthesis pathways, including genes encoding key enzymes and regulatory transcription factors, were uncovered primarily by characterizing cuticle mutants in arabidopsis, rice, maize, barley and wheat [9,10,11,12,13,14,15,16,17,18,19,20,21]. As many as 47 cuticle-associated genes were discussed in the review of Yeats and Rose [2]. At least 21 genes of arabidopsis were predicted to encode b-ketoacyl-CoA synthase [2] that catalyzes the initial condensation step in the elongation pathway of VLCFA biosynthesis. In maize (*Zea mays*), at least 18 loci (the GLOSSY or GL loci) have been found to affect the quantity and/or the composition of cuticular waxes on the surface of seedling leaves (in: [25]).

Despite considerable knowledge about the genetic basis of wax formation in various species, rye remains almost unrecognized in this area of knowledge. With regard to the literature on rye wax genes, references to the location of only two genes on low density genetic maps can be found [43,44]. Our study, aimed at mapping the allele conditioning the nonglaucous appearance derived from the rye cultivar Karlikovaja Zelenostebelnaja, showed that the studied gene was mapped for the first time because it was linked to group 2R, while the previously tested genes were located on chromosome 4R and 7R [43,44].

### 3.1. Mapping Procedure with ML Methods Support

Molecular markers are assigned to linkage groups on the basis of a logarithm of odds (LOD) value, which refers to the ratio of the probability that two loci are linked with a given recombination value over a probability that the two are not linked. The first genetic maps composed of a relatively small number of markers were constructed with use a critical LOD (linklod) value of three as the minimum threshold value in order to decide whether or not loci were linked, which indicates that linkage is 1000 times more likely than no linkage. Modern, high-density maps, based on thousands of marker data, require a significant increase in the level of linklod. Establishing this value, and determining number of linkage groups, is not a straightforward task. Loci on different chromosomes may appear to be linked by chance and, on the other hand, two or more linkage groups can be obtained for each chromosome, which results in a total number of linkage groups higher than the haploid chromosome numbers [53]. In practice, different LOD values for each experiment have to be tested until in order to establish satisfactory linkage groups equal to haploid chromosome number. The correctness of marker grouping depends on many factors, including the type and size of population and the number and type of marker data (dominant or codominant).

Increasing the size of the mapping population is a good approach to improve the reliability of linkage group creation, but it must also be economically feasible. The main purpose of our research was to map specific gene encoding wax and to preselect markers closest to the studied gene. That is why about 180 genotypes were targeted for genotyping, which was a balance between map quality and the economic aspect of research.

Adopting the right LOD level in a mapping procedure is frequently problematic. In our study, although the linklod at which the two records of wax segregation data (co- and dominant) remained coupled was high (36), it resulted in a map design with an unsettled order relative to the reference map [56]. In addition, mapping of the *wax* region with several sets with different numbers of wax-flanking loci resulted in instability of the marker order. A way to increase the reliability of the map was to reduce the linkage group by further increasing the LOD up to 50. This, in turn, separated the two wax segregations and caused the loss of all DArTseq-Silico loci, suggesting that this criterion was too restricted. To improve the mapping quality, an additional selection of markers was made using three ML methods. Both DArTseq, SNP and Silico were identified in this procedure. Loci selected as flanking in a classical way and indicated by three ML methods, were used for final mapping of the wax region.

This combined mapping strategy led to the construction of a stable map and detected three potential candidate genes for forming a cuticular wax in the 2 cM region from the phenotypic marker. Two of them (DArTseq Silico) were detected only as a result of the use of ML methods, and the third (3362575_18:C>T) was selected in both approaches, which indicates the usefulness of ML models in choosing a group of markers linked to a specific gene, during the mapping procedure.

One of DArTseq Silico (3585843) seems to be a strong functional marker candidate for the ABC transporter gene. The next two annotated markers were even more closely linked to glaucousness but their homology to known genes and their role was not so obvious. They can be considered as putative genes coding proteins of enzymatic character.

### 3.2. Candidate Gen for ABC Transporter G Family Member

DArTseq 3585843 was considered a functional candidate for the ABC transporter gene marker because it showed 100% sequence similarity to the sequence of predicted rice ABC transporter G family member 11-like (LOC102717335) mRNA (Table 4). The expression of this candidate gene in qPCR (Figure 5) showed differences between the glaucous and the nonglaucous parent of mapping population BK2.

The ATP-binding cassette (ABC) transporter’s role in transporting wax precursors has already been reported in the literature. ABC transporter proteins belong to a large, diverse and ubiquitous superfamily involved in the active transport of a wide range of molecules across membranes. Arabidopsis and rice genomes encode 130 and 132 members, respectively, and their encoded proteins are categorized into different subfamilies [80]. Recently released plant genome sequences allowed the identification of 803 ABC transporters in four vascular plants and 76 transporters in a green alga, by comparing them with those reannotated in *Arabidopsis thaliana* and the yeast *Saccharomyces cerevisiae* [81]. The ABCG is the largest one among eight (A-I) plant subfamilies [40,81]. Particular classes of multiple ABC transporters participate in the transport of different lipids, including FAs, waxes, and sterols [82].

By analyzing mutants in arabidopsis, ABCG half-transporters required to accumulate both cutin and wax at the cell surface were identified. AtABCG11 is responsible for the formation of cutin, through transport of lipid precursors from epidermis to cuticula, and the abcg11/wbc11/desperado/cof1 mutants displays reduced cutin and wax levels [34,35,83,84,85]. ABCG11 has a broad substrate specificity for a variety of structurally diverse cuticular lipids, and can directly transport cuticular lipids [34,35,83,84,85].

ABCG were also considered to be essential components of the plant immune system [86]. This is consistent with the observation that epicuticular waxes play roles in plant-insect interactions [87,88]. This aspect makes our putative gene an interesting research object in the context of rye resistance to pathogens.

### 3.3. Putative Candidate Gene for Fatty Acid Desaturase (FAD)

DArTseq 3889647 was the most closely linked to the wax locus. Its sequence showed homology mostly to uncharacterized, unannotated sequences of rye-related species. Among them was the complete sequence of *Oryza sativa* genomic DNA, chromosome 4, BAC clone (Table 4), which was consistent with the collinearity rule. Comparative genomic studies indicated that rye chromosome 2R is a counterpart of, among others, rice chromosome Os04 [55].

The blasting procedure suggested that the DArTseq 3889647 sequence was 90% similar to predicted the glycine soja long chain-fatty acid- AMP ligase FadD28-like (LOC114378589) mRNA sequence (Table 4). FADs are widely distributed throughout the biological world. They are present in all groups of organisms, namely bacteria, fungi, plants and animals, and play a key role in the maintenance of the proper structure and functioning of biological membranes [89]. Their main function is to remove hydrogen from hydrocarbon chains of FAs in the biosynthesis of unsaturated FAs to synthesize C-C double bonds at specified positions. Although the reports of FadD28 were not found in the plant literature, the other FAD family enzymes are common among plants and are involved in the metabolism of FAs. Long-chain-FA--AMP ligase FadD28 occurs in mycobacterium and catalyzes the activation of long-chain FAs (C22–24) as acyl-adenylates (acyl-AMP), which are then transferred to the multifunctional polyketide synthase Mas for further chain extension (UniProtKB-9WQ59: FAA28_MYCTU).

In current genomic studies of plants, 23 full-length FAD genes have been identified in cucumber (*Cucumis sativus* L.) through database searches. They were distributed on all seven chromosomes and two additional scaffolds [90]. In an upland cotton (*Gossypium hirsutum* L.) study, 39 full-length FAD genes, based on database searches, were identified in tetraploid and were phylogenetically clustered into four subfamilies. Genomic localization revealed that 34 genes were mapped on 22 chromosomes, and five genes were positioned on the scaffold sequences [91].

Recently, a total of 20 full-length desaturase genes were identified from the rice genome. The rice desaturase genes were phylogenetically classified into six subfamilies with the arabidopsis counterparts FAB2, FAD2, FAD3/7/8, FAD6, DES1 and SLD1, and distributed on ten of 12 chromosomes. According to genome collinearity order, rye chromosome 2R is a counterpart of rice chromosomes 4, 6 and 7 [55]. Rice chromosome Os07 contains four desaturase genes; chromosome Os04 has one member [92]. Sequences of these five desaturases have a low level of similarity to DArTseq 3889647 (data not shown), which undermines our hypothesis. However, among twenty rice desaturases, nine members were expanded via chromosomal tandem or segmental duplications [92]. Since rye is separated from rice by a long evolutionary path (probably over 60 million years) and the much larger rye genome may have many genomic sequences with no homology to rice, it is possible that the rye gene is the result of duplication and changes of one of the desaturases. The second possible explanation is that the rye putative FadD28 gene is derived from bacterial DNA built into the rye genome. Another possibility is that we are dealing with an unknown gene unrelated to FAD.

### 3.4. Putative Candidate Gene for Leucine-Rich Repeat Receptor-Like Protein Kinase (LRR RLK)

The marker 3362575_18:C>T is at a distance of less than 1 cM from the *wax* locus. Its sequence shows high similarity (96%) to predicted *Aegilops tauschii* subsp. *tauschii* probably inactive LRR-RLK At5g06940 (LOC109756806) mRNA.

Receptor-like kinases (RLKs) containing leucine-rich repeats (LRRs) are members of the family of membrane-localized receptor kinases containing extracellular leucine rich repeat regions. LRR RLK acts as both signal receptor and signal transducer in ligand-mediated communication between cells. The function of many LRR RLKs is still unknown. However, there are some papers describing the relationship between LRR RLK and brassinosteroids (BRs). BRs are perceived by the LRR RLK, binding to a subdomain of these repeats, thereby initiating intracellular signal transduction via activating a kinase cascade beginning with receptor autophosphorylation and culminating in altered gene expression [93]. BRs belonging to lipidic plant hormones may affect the FA composition of cell membranes [94]. Various possible directions of BRs’ action on a membrane can be noted. The first is the influence of BRs on the FA composition towards an increased proportion of unsaturated FAs. This suggests the involvement of BRs in the biosynthesis of FAs, or FA transport, and incorporation into cell membranes. BRs, as some other sterols, may enter the cell membrane directly and modify its properties through, for example, increasing the distance between FA chains, which also may improve the functioning of the membrane under unfavorable temperature conditions [94].

The RLK-BRs-FA relationship indicated here suggests a possible gene candidate effect on the formation of a normal wax coat.

The close proximity of three discussed loci linked to wax on the genetic map may suggest that these genes, selected as candidates for participation in wax metabolism, have a similar function and, therefore, are grouped in a metabolic gene cluster. Recent progress in research on the wheat wax inhibitor has shown that the *W1* locus constitutes a β-diketone biosynthetic gene cluster including diketone metabolism-polyketide synthase (DMP), diketone metabolism-P450 CYP709J4 (DMC) and diketone metabolism-hydrolase/carboxylesterase (DMH) [33].

### 3.5. Summary

Rye is a small grain cereal closely related to wheat, barley and triticale. All these crops contain very large genomes (5–17 GB). Sequencing of so abundant DNA sets has been a great challenge. The first significant approach done for *S. cereale* in 2017 [57] released 1.3 million scaffolds covering approximately 30–35% of the rye genome. This database was applied in our study, allowing indication of putative genes for wax synthesis in rye. Unfortunately, we have not succeeded in the indisputable targeting of the gene of interest. Recently released new data on the wheat genome sequence [95], and the more advanced version of rye genome sequence [96], opened new opportunities in candidate gene identification. Indication of the *ScGA2ox12* gene as a candidate for determinant of the dominant type of dwarfing named *Ddw1* in rye [97] can be showed as a case in point. Thus, the further analyses should tend to combine the current DNA-sequence databases with novel methods of computational analyses based on machine learning.

Although knowledge about the genetic basis of wax formation is still expanding, understanding cuticle biosynthesis at the molecular level remains incomplete. Progress in identifying these pathways is necessary to complete the knowledge and to enable selective modification of cuticle properties to improve agricultural productivity. Even for intensively studied plant species, there is a need to continue and extend current knowledge about cuticular wax metabolism processes. To better understand the molecular mechanism of cuticular wax biosynthesis, more putative genes that are thought to participate in the process need to be characterized.

Scientific progress depends on interdisciplinary analyses and requires the integration of bioinformatic analyses of DNA sequences with our knowledge at the biochemical and molecular level. Machine learning is one of the options that can help humans to integrate these areas of research activity.

## 4. Material and Methods

The plant material for this study was a rye mapping population named BK2, which was the F_2_ generation of an interline hybrid between inbred lines AK1 and AKZ. The line AK1 (S_10_) was obtained by selfing one dwarf plant found among offspring of the intergeneric hybrid *S. cereale* cv Amilo × *Dasypyrum villosum*. The second parent line AKZ, originates from *S. cereale* cv Karlikovaja Zelenostebelnaja, the old Russian cultivar carrying the unknown gene responsible for wax formation abnormalities. Unlike typical rye plants, individuals with this gene look like they have no blue coating on their ears, leaves and stems (Figure 1). This kind of phenotype is called nonglaucous or waxless.

Experimental trials were conducted in the field of the West Pomeranian University of Technology in Szczecin (53.45° N, 14.53° E) in two vegetation seasons (2016–2017 and 2017–2018). Meteorological data for the years of conducting experiments are shown in the Table S8. All applicable ethical standards were followed. On 20 September 2016, 300 grains of BK2-F_2_ were sown at a spacing of 20 × 20 cm. In the spring, before flowering, two ears of each of 271 surviving plants were covered with cellophane casings for self-pollination. After harvesting, the sheltered ears were used for measurements and the grains collected from them to prepare a field experiment the following season. The studied generation BK2-F_3_, was the offspring of F_2_ individuals, each represented by 8–16 plants grown in one to two rows.

Phenotyping of BK2 was done on plants of the F_2_ and F_3_ generations. The assessment of the presence of a wax coating was done visually on the whole plants in the stem elongation phase. Obtaining codominant segregation of glaucousness was possible by assessing the F_3_ progeny of each individual F_2_ plant. Glaucous plant of F_2_ was considered the dominant homozygote if at least 12 of its F_3_ offspring were glaucous in the absence of any nonglaucous plant.

In addition to wax, the following traits were studied on mature plants: number of tillers (TN), plant height (PH), length of main spike (SL), number of spikelets per main spike (SNPS), number of grains per main spike under isolation (GNPS) and weight of grains per main spike (GWPS). Compactness of the main spike (CT), determined as the number of spikelets per 10 cm, and average thousand grain weight (TGW), were calculated based on counted/measured parameters. In the case of the F_3_ generation, the height of all offspring plants was measured (max. 16 per genotype) and the ear / grain parameters were assessed for three isolated ears from different plants of a given genotype (the progeny of each F_2_ plant). Flowering date (FD) was given as the number of days from May 1st. It was evaluated for the F_3_ generation as the day on which half of the plants in a single row bloomed (visible anthers).

Genotyping was conducted on the DNA extracted from the young leaves of F_2_ plants using DNeasy Plant Mini Kit (Qiagen, Hilden, Germany) according to the manufacturer’s instructions. A set of 184 individuals, randomly chosen from 271, and two parental lines, were sent to the Diversity Arrays Technology Pty Ltd. (Canberra, Australia) for genotyping by sequencing (http://www.diversityarrays.com/). Received segregations of DArTseq were used to localize the *wax* locus on the newly developed genetic map of population BK2.

Total RNA for expression analyses was isolated from leaves in the stem elongation phase (BBCH 34–37) using the Qiagen RNeasy Plant Miniprep Kit (Qiagen, Hilden, Germany) according to the manufacturer’s procedure. The leaves were frozen in liquid nitrogen and homogenized using TissueLyser II (Qiagen, Hilden, Germany). The quantity and quality of RNA was assessed using an Epoch Microplates Spectrophotometer (BioTek Instruments, Inc., Winooski, VT, USA). For all samples, the 260/280 ratio fluctuated around 2.0. 2 µg of total RNA was used for reverse transcription which was carried out immediately after RNA extraction using the QuantiTect ReverseTranscription Kit (Qiagen, Hilden, Germany).

Real-time PCR was performed in a RealPlex4 Mastercycler (Eppendorf, Hamburg, Germany) using GoTaq^®^ qPCR Master Mix (Promega, Madison, WI, USA), 0.3 μM of ach primer (Table 5) and 1 μL cDNA templates in a final volume of 20 μL. The reaction was carried out starting from 120 s of activation at 95 ° C followed by denaturation for 15 s at 95 °C and annealing/elongation at 60 °C for 60 s. The reactions were carried out in 40 cycles. Melting curve analysis (60–95 °C) was performed at the end of each PCR thermal profile. A negative control for each set of primers was performed to ensure amplification specificity. 

*ABCG11* primers (Table 5) were developed based on a consensus sequence created using the DArTseq 3585843 sequence and homologous sequences of wheat (>AK331244.1 *Triticum aestivum* cDNA, clone: WT007_A05, cultivar: Chinese Spring) and barley (>AK355515.1 *Hordeum vulgare* subsp. *vulgare* mRNA for predicted protein, complete cds, coding sequences, clone: NIASHv1021O01), which were selected indirectly as homologous to >XM_006652486.2 predicted: *Oryza brachyantha* ABC transporter G family member 11-like (LOC102717335) mRNA. A Bio-Edit 7.2 computer program [98] was used to establish consensus sequence and Primer 3 [99] to create primers.

Stability of three reference genes: actin (ACT), glyceraldehyde 3-phosphate dehydrogenase (GAPDH) and RNase L inhibitor (RLI), was evaluated using geNorm and NormFinder algorithms. *ScABCG11* activity was assessed relative to the ACT and GAPDH. The relative gene expression was calculated using GeneEx 7.0 (MultiD Analysis) software. The qPCR reaction was performed for three biological and three technical replicates.

Linkage analysis was made using the JoinMap 5.0 package (www.kyazma.nl). MLH mapping was used to construct a map of the whole chromosome 3R, and the RM procedure (Kosambi function) was used for mapping a narrowed region with markers linked to *wax* locus.

Statistical analysis. The statistical relationship between the segregation of phenotypic traits of the mapping population was analyzed with the Student’s *t*-test using STATISTICA v. 12.0 software (http://www.statsoft.com). Significance of differences between parental lines (phenotypic traits and expression level in qPCR) was established by employing the nonparametric Kruskal-Wallis test.

The ML models and selection process were realized in a Python environment using inter alia Scikit-learn and XGBoost libraries [100]. Data were randomly split into a 60% set to train models and a 40% test-set used to evaluate classification metrics. Three independent classifiers were trained: LR, and two ensembled models, random forest and extra boosted trees (XGBClassifier). The crucial models’ hyperparameters (number of trees in ensemble classifiers and penalty strength in LR and XGBClassifier) were set using the RandomizedSearchCV method using 100 iterations on specific uniform distributions. The impact of each of the randomly selected parameters on classification accuracy was measured. Parameters that assured the highest accuracy of the models were used as final ones. Feature (marker) selection was performed using the SelectFromModel method which removes features considered as less or noninformative when the importance of the feature is below a specific threshold (mean of all feature importances). Each marker indicated as informative by all of the models was considered as significant.

Models were evaluated using classification report including accuracy (fraction of correctly predicted labels), precision (ratio of true positives divided by sum of true positive and false positive predictions), recall (ratio of true positive divided by sum of true positive and false negative predictions) and f1 metrics (harmonic mean of precision and recall), for each class [100]. A t-distributed stochastic neighbor embedding (t-SNE) algorithm was used to visualize high-dimensional data in a two-dimensional space before visualization data was standardized according to rules described in the scikit-learn documentation [100]. The analysis described above is available on GitHub (https://bit.ly/3b4sBNI).

## Figures and Tables

**Table 1:** The significance of differences between the group of waxy (**1**) and waxless (**2**) plants of mapping population BK2, demonstrated using the *t*-test.

Trait	Generation	Mean		Standard Deviation		Sample Number		*t*-Statistic	Degrees of Freedom (df)	Probability Level (*p*-Value)
		1	2	1	2	1	2			
PH *	F_2_	116.10	127.07	29.10	26.34	200	68	−2.75	266	0.006
	F_3_	96.40	102.25	26.19	28.44	248	128	−1.99	374	0.047
TN	F_2_	3.83	3.38	2.11	1.60	202	69	1.62	269	0.106
	F_3_	3.53	3.36	1.88	1.65	248	128	0.88	374	0.380
SL	F_2_	10.45	10.21	1.74	1.72	196	67	0.97	261	0.333
	F_3_	9.27	9.23	1.40	1.29	245	126	0.30	369	0.768
SNPS *	F_2_	32.84	34.27	4.84	4.97	196	67	−2.07	261	0.040
	F_3_	30.75	32.27	4.32	3.79	245	126	−3.34	369	0.001
CT *	F_2_	31.84	33.93	4.54	4.46	196	67	−3.27	261	0.001
	F_3_	33.41	35.34	3.73	4.38	245	126	−4.43	369	0.000
GNPS	F_2_	39.14	39.90	25.68	27.20	196	67	−0.20	261	0.839
	F_3_	21.21	21.68	17.60	17.32	245	126	−0.25	369	0.807
GWPS	F_2_	1.36	1.36	0.60	0.69	159	55	−0.04	212	0.965
	F_3_	0.64	0.57	0.39	0.41	182	106	1.41	286	0.160
TGW	F_2_	28.16	27.41	6.43	7.07	159	55	0.74	212	0.463
	F_3_	22.21	21.80	6.72	6.21	182	106	0.51	286	0.611
FD *	F_3_	28.61	29.01	0.87	0.89	133	50	−2.73	181	0.007

PH—plant height (cm), TN—tiller number, SL—spike length, SNPS—spikelet number per spike, CT—spike compactness (spikelet number per 10 cm), GNPS—grain number per spike, GWPS—grain weight per spike (g), TGW—thousand grain weight (g), FD—flowering data (days from May 1st), * statistically significant differences.

**Table 2:** DArTseq statistic in rye mapping population BK2-F_2_.

	Silico	SNP	Total
incomplete data	447	2914	3361
monomorphic	2630	1267	3897
polymorphic	25812	6545	32357
	including		
1R *	917	191	1108
2R *	927	208	1135
3R *	977	152	1129
4R *	873	188	1061
5R *	1086	169	1255
6R *	1165	231	1396
7R *	789	183	972
mean per chromosome	962	189	1151
unassigned **	19152	5070	24222

*/** DArTseq assigned/unassigned to chromosomes based on literature data [56,57].

**Table 3:** DArTseqs indicated by three machine learning (ML) algorithms as important for distinguishing between waxy and waxless plants. Coefficient values are draw out directly from models (in the case of logistic regression (LR) absolute values of coefficients were used). Impact value is the sum of coefficients.

Marker	Assignment to 2R	LR Coefficient	Random Forest Coefficient	XGBoost Coefficient	Impact	Distance from *Wax* Locus [cM]	Annotation
3591025	A	0.636	0.017	0.825	1.478	1.691	-
3593882	B	0.553	0.022	0.024	0.599	unmapped	+
3578307_27:A>G	B	0.413	0.013	0.013	0.439	1.944	-
3889647	A	0.285	0.025	0.035	0.345	0.088	+
3908692_28:C>T	B	0.315	0.014	0.008	0.337	1.097	-
3362575_18:C>T	B	0.192	0.014	0.086	0.293	0.81	+
4485942_42:T>G	B	0.192	0.002	0.069	0.263	0.86	-
3597393_10:T>G	B	0.062	0.005	0.162	0.228	unmapped	-
3358122	A	0.162	0.014	0.019	0.195	7.162	-
3585843	A	0.169	0.015	0.01	0.193	2.056	+
3341848	A	0.064	0.002	0.002	0.067	6.917	+
4092788_55:G>A	B	0.044	0.004	0.009	0.058	22.593	+
3750485	B	0.022	0.01	0.002	0.034	unmapped	-

**Table 4:** Annotations of markers most strongly linked to *wax* locus (<3 cM) found in the NCBI database.

Marker	Description	Total Score	*E* Value	Identity	Accession
3889647 ^1^	Predicted: glycine soja long-chain-fatty-acid--AMP ligase FadD28-like (LOC114378589), mRNA	71	1E–09	90%	XM_028337226.1
3362575_18:C>T ^1,2^	Predicted: *Aegilops tauschii* subsp. *tauschii* probably inactive leucine-rich repeat receptor-like protein kinase At5g06940 (LOC109756806), mRNA	112	4E–22	96%	XM_020315644.1
3585843 ^1^	Predicted: *Oryza brachyantha* ABC transporter G family member 11-like (LOC102717335), mRNA	128	2E–26	100%	XM_006652486.2

^1^ marker pointed with use ML; ^2^ marker pointed with use MLH (JoinMap 5.0).

**Table 5:** Primers used in qPCR for amplification of reference genes and studied gene *ScABCG11*.

Gene	Primer Pair	Sequence 5′–3′
Actin (ACT)	ACT Fw	AAGATGGGACGTCTTGATGG
	ACT Rev	GGATCTTCATCGGCATCACT
Glyceraldehyde 3-phosphate dehydrogenase (GAPDH)	GAPDH Fw	AGATGCCCCTATGTTTGTGG
	GAPDH Rev	GTGGTGCAGCTAGCATTTGA
RNase L inhibitor (RLI)	RLI Fw	TTGAGCAACTCATGGACCAG
	RLI Rev	TGCTTTCCAAGGCACAAACAT
ATP binding cassette transporter, subfamily G (*ABCG11*-like)	ABCG_F_1297	GGTGATGGATTCAAGGGGCA
	ABCG_R_1382	CGCGCGACATGTTGATGAAT

## Supplementary Materials

The following are available online at https://www.mdpi.com/1422-0067/21/20/7501/s1, Table S1: Segregations of DARTseqs in rye mapping population BK2, assigned to 2R based on literature; Table S2: Segregations of DARTseqs in rye mapping population BK2, not assigned to chromosomes based on literature; Table S3: Segregations of DARTseqs in rye mapping population BK2, selected to map 2R; Table S4: The reference map of chromosome 2R of rye RIL-S population [56]; Table S5: Map of 2R of rye BK2 population constructed of 883 loci linked to wax at LOD 50 using maximum likelihood algorithm; Table S6: Loci connected with wax segregation, selected based on three machine learning methods; Table S7: Wax locus region map constructed using 31 loci (17 nearest flanking loci from whole BK2 map and selected 13 loci pointed out in three machine learning methods) using a regression mapping algorithm; Table S8: Meteorological data for the years of conducting experiments at the field of the West Pomeranian University of Technology in Szczecin (Poland).

## Abbreviations

ABC	ATP-binding cassette
BRs	brassinosteroids
CER	eceriferum
cM	centimorgan
CT	compactness of the spike
cv	cultivar
DArTseq	sequencing-based diversity array technology
FA	fatty acid
FAD	fatty acid desaturase
FD	flowering date
GBS	genotyping by sequencing
GNPS	number of grains per spike
GWPS	weight of grains per spike
LOD	logarithm of odds
linklod	critical LOD
LR	logistic regression
LRR	leucine-rich repeat
ML	machine learning
MLH	maximum likelihood
NCBI	National Center of Biotechnology Information
PH	plant height
RIL	recombinant inbred line
RLK	receptor-like protein kinase
RM	regression mapping
SL	spike length
SNPS	number of spikelets per spike
TGW	thousand grain weight
TN	tiller number
VLCFA	very long-chain fatty acid
